# Vortex Formation Times in the Glottal Jet, Measured in a Scaled-Up Model

**DOI:** 10.3390/fluids6110412

**Published:** 2021-11-15

**Authors:** Michael Krane

**Affiliations:** Applied Research Laboratory, Penn State University, State College, PA 16804, USA;

**Keywords:** phonation, glottal jet, instability, voiced sound production

## Abstract

In this paper, the timing of vortex formation on the glottal jet is studied using previously published velocity measurements of flow through a scaled-up model of the human vocal folds. The relative timing of the pulsatile glottal jet and the instability vortices are acoustically important since they determine the harmonic and broadband content of the voice signal. Glottis exit jet velocity time series were extracted from time-resolved planar DPIV measurements. These measurements were acquired at four glottal flow speeds (*u*_SS_ = 16.1–38 cm/s) and four glottis open times (*T*_o_ = 5.67–23.7 s), providing a Reynolds number range *Re* = 4100–9700 and reduced vibration frequency f* = 0.01–0.06. Exit velocity waveforms showed temporal behavior on two time scales, one that correlates to the period of vibration and another characterized by short, sharp velocity peaks (which correlate to the passage of instability vortices through the glottis exit plane). The vortex formation time, estimated by computing the time difference between subsequent peaks, was shown to be not well-correlated from one vibration cycle to the next. The principal finding is that vortex formation time depends not only on cycle phase, but varies strongly with reduced frequency of vibration. In all cases, a strong high-frequency burst of vortex motion occurs near the end of the cycle, consistent with perceptual studies using synthesized speech.

## 1. Introduction

This article describes the timing of instability vortex formation on the unsteady jet formed between two moving walls using data previously published [1,2]. The model used for the experiment was sized to mimic the fluid dynamics of human phonation (i.e., the production of voiced speech sounds by the fluid-structure interaction between the vocal folds and flow between them). The flow through the glottis (the space between the vocal folds) develops the glottal jet, which is modulated by the opening and closing of the glottis during each vibration cycle. Glottal jet instability vortices are believed to contribute to the “breathy” component of voiced sound.

Voiced speech sounds contain both strong harmonic content and broadband content due to the glottal jet. It is believed that the broadband content is due to glottal jet turbulence [3] or, more precisely, glottal jet instability vortices, whose timing is less phase-coherent to the motion of the vocal folds than the longer time-scale pulsations of the glottal jet [4]. Indeed, whispering is performed entirely by voluntarily immobilizing the vocal folds and forming a steady glottal jet [3,4]. Previous work [1,2] made note of these glottal jet vortices and that their effect on the exit velocity waveform involved short-time scale, large-amplitude fluctuations, but did not go further in characterizing the timing of these vortices.

Hermes [5] further showed an intriguing relationship between the periodic and broadband acoustic components of the voice. He suggested that the broadband contribution to the voice sound source waveform occurs primarily during a short “burst” at a particular phase, late in the cycle, rather than over the entire time the glottis is open. This conclusion was based on listening tests with synthesized voice signals wherein the phase of the high-frequency noise burst was varied. When the burst appeared at a particular phase, the voice signal was perceived as “natural”, whereas if the burst were added at other phases, the broadband sound was perceived separately as the periodic voice and a separate broadband background. Similar broadband “burst” behavior was observed using wavelet analysis of radiated sound from a periodically varying orifice that was built to glottal dimensions and shape [6]. Coker, et al. [7] showed that this particular delay in broadband burst occurrence is consistent with convection of the glottal jet past a vocal tract wall protuberance such as the epiglottis. None of the authors in [5–7] examined glottal jet vorticity in detail to make clear the connection between glottal jet vortices and the broadband acoustic behavior they observed.

Aeroacoustic theory [8–14] shows that the principal source of sound in phonation is vocal fold drag. Recent experimental results show that vocal fold drag is essentially equivalent to transglottal pressure force [15–17], which is proportional to the square of the jet velocity exiting the glottis. Vocal fold drag is related to glottal jet vorticity, as described in detail in refs. [4,8,15,18]. Vortex sound theory also shows that vocal fold drag is determined by time-varying glottal jet structure, in terms of the strength and path of jet vorticity, and the instantaneous shape of the vocal folds.

There are then several issues regarding glottal jet vortex timing that are relevant for voiced sound production: (1) how phase-coherent are the vortices with the vocal fold vibration cycle? If they are not, they contribute to the broadband component of the voice signal. (2) Does the jet structure show any evidence of a concentrated burst of broadband energy at a particular phase of the vibration cycle, particularly near the end of the cycle?

As noted above, the data used for the present analysis comes from the time-resolved DPIV measurements of a model glottal jet presented in [1,2]. Others [19–28] have also performed DPIV measurements of glottal flow, but these measurements did not combine the following: (1) sufficient time resolution; (2) a focus on the details of jet instability vortex structure, especially with regard to contributions of jet instability vortices to the broadband component of the jet velocity; and (3) a range of flow speeds and cycle frequencies.

This article, then, focuses on characterizing the timing of instability vortices on the glottal jet, and how this timing varies with Reynolds number and reduced frequency of vibration. The implications of these findings for voiced sound production will then be discussed.

## 2. Materials and Methods

**Figure 1.**
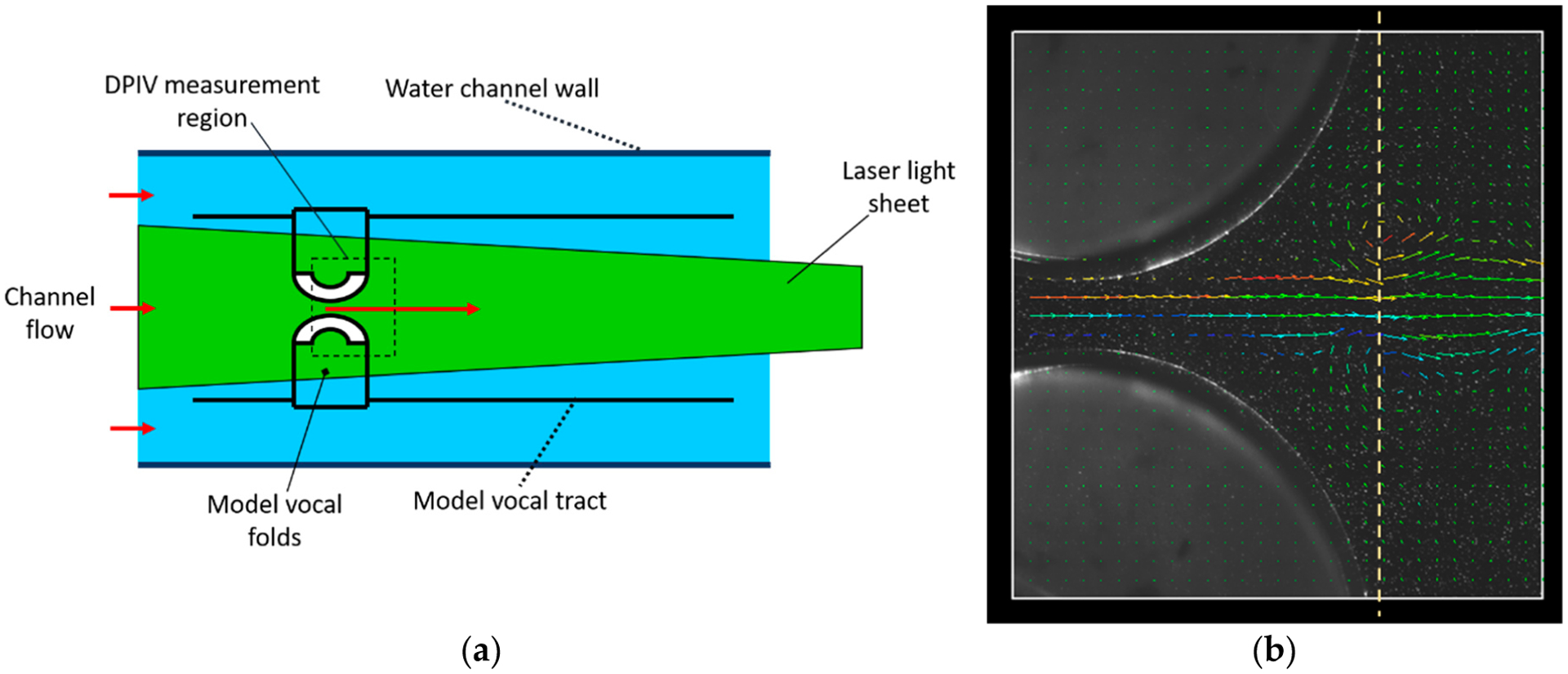
(**a**) Schematic of experiment [1,2] that provided measurements analyzed in this work. Vocal tract model is immersed in a water channel. Flow around channel pressurizes subglottal region, forcing flow through the vocal folds when glottis is open. Vocal folds open and close for a single cycle, during which DPIV measurements are performed in the region shown. (**b**) Still of DPIV measurements, superimposing video image and flow velocity vector field, showing passage of a glottal jet instability vortex through the glottis exit, indicated by the vertical dotted line.

The measurements used in the analysis presented here were acquired from flow of water through a scaled-up idealized constriction that mimics the motion of the human vocal folds during phonation. By scaling up the size by a factor of 10, and using water as the working fluid (kinematic viscosity ratio of 1/15), the model time scales were 1500 times life scale. This enabled time resolved flow measurements, even with the 30 Hz double-shutter digital video cameras used for these measurements. The larger field size also improved spatial resolution relative to scattering particle sizes. Full details are given in [1,2]. Figure 1a shows a schematic of the flow geometry. Figure 1b shows a still image superimposing the raw image and PIV-estimated velocity field, at the instant when a vortex pair passes through the exit plane.

To quantify the instability vortex behavior, this work uses waveforms of the velocity at the exit plane indicated in Figure 1b. Since the glottal jet path varies from cycle to cycle [1,2,16,17], and since the location of the glottal jet on the exit plane is indicated by the location of the instantaneous velocity maximum, we use the waveform of maximum exit velocity, *u*_max_, for the analysis. As further discussed below, the correspondence between high-frequency content on the glottal jet velocity waveforms at the glottis exit and the passage of jet instability vortices past that location [1,2], permits straightforward characterization of vortex formation time.

## 3. Results

### 3.1. Cases Studied

Table 1 lists the cases studied. As detailed in [1,2], measurements were performed for a single cycle of wall motion. Two parameters were set for each measurement: The first is the steady state tunnel speed, which is quantified by the flow speed *u*_SS_ measured in the glottis, with the glottis held open to maximum width *h*_max_. The second parameter is the time *T*_o_ for the glottis walls to open and close. Note that for comparison purposes, we arbitrarily set the vibration cycle period to 2*T*_o_—in other words, the vocal folds are open for half the vibration cycle period. Four cases were acquired for *u*_SS_ = 28 cm/s, with *T*_o_ ranging from 5.67 s to 23.7 s, and four cases were acquired for *T*_o_ = 6.53 s, with *u*_SS_ ranging from 16.1 cm/s–38 cm/s. Note that the *u*_SS_ = 28 cm/s, *T*_o_ = 6.53 s (*Re* = 7200, f* = 0.035) case is common to both sets. Also indicated in Table 1 are the maximum glottis gap opening *h*_max_ for each case, the reduced vibration frequency f* = *L*/(*u*_SS_·2*T*_o_), where *L* = 15.7 cm is the glottis length, the Reynolds number *Re*_h_ = *u*_SS_·*h*_max_/ν, the number *N* realizations acquired each condition, and the equivalent life scale voice frequency *f*_life_ = 1500/(2*T*_o_).

### 3.2. Exit Velocity Behavior

**Figure 2.**
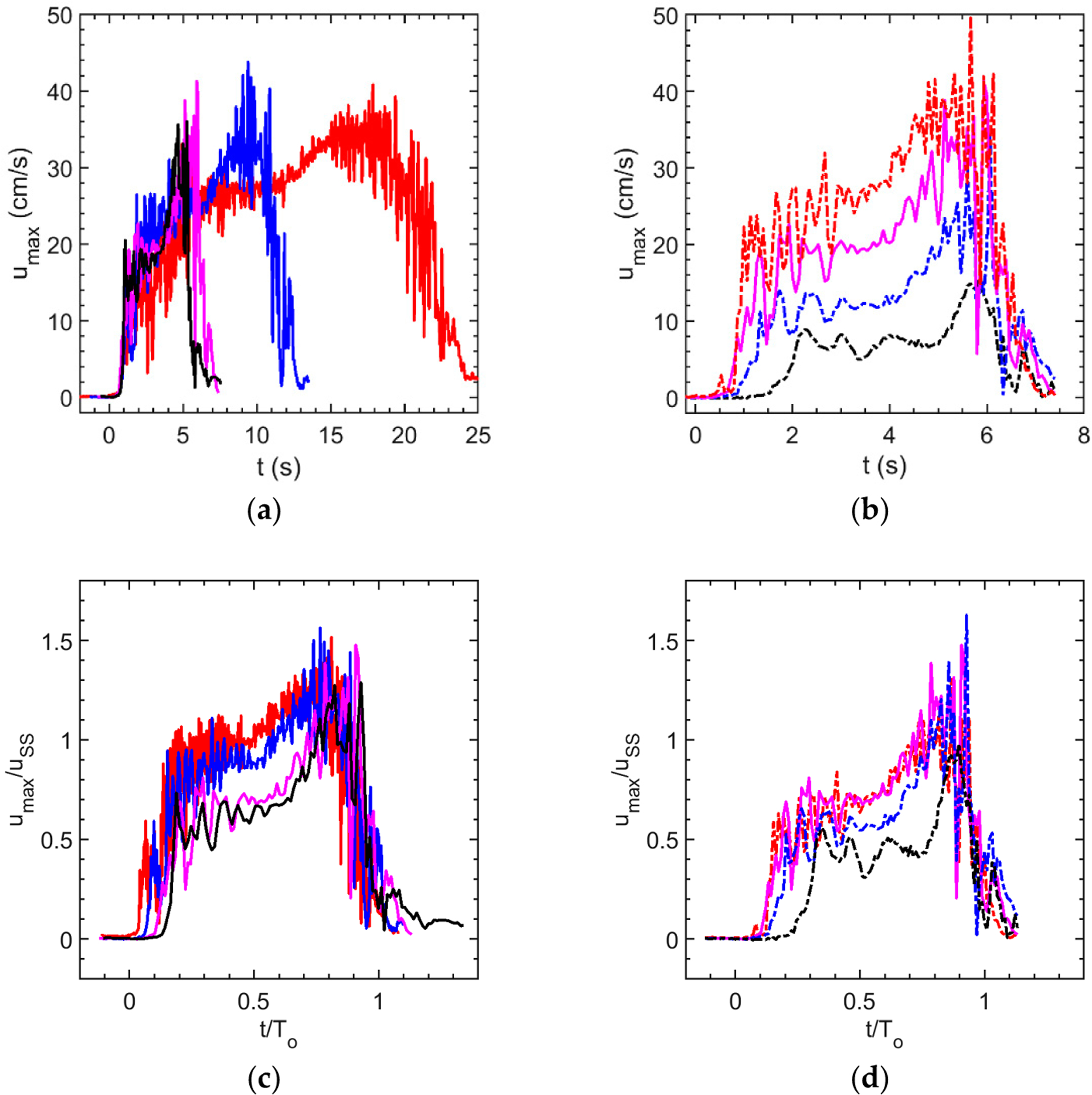
Exit velocity u_max_ waveforms. Each panel shows a single realization for each of the cases studied. (**a**) *u*_max_ vs time for *u*_SS_ = 28 cm/s cases: red solid line, *T*_o_ = 23.7 s; blue solid line, *T*_o_ = 12.3 s; magenta solid line, *T*_o_ = 6.53 s; black solid line, *T*_o_ = 5.67 s. (**b**) *u*_max_ vs time for *T*_o_ = 6.53 s cases: red dash-dot line, *u*_SS_ = 38 cm/s; magenta solid line, *u*_SS_ = 28 cm/s; blue dash-dot line, *u*_SS_ = 21.3 cm/s; black dash-dot line, *u*_SS_ = 16.1 cm/s. Note that the *T*_o_ = 6.53 s, *u*_SS_ = 28 cm/s case (magenta solid line) appears in both (**a**,**b**). (**c**) Same data as in (**a**), but axes nondimensionalized (same legend). (**d**) same as (**b**), but axes nondimensionalized (same legend). Refer to Table 1 for corresponding Reynolds number and reduced frequency.

Before focusing on instability vortex timing, let us first examine the overall behavior of the jet through waveforms of maximum jet speed at the glottis exit. Figure 2 shows these waveforms, showing one realization each for the cases listed in Table 1. Figure 2a shows jet speed vs. time where the tunnel speed was held constant, but the cycle period *T*_o_ was varied (*u*_ss_ constant, *T*_o_ varying). Figure 2b shows the other set of cases, where the tunnel speed was varied, but *T*_o_ was held constant (*u*_ss_ varying, *T*_o_ constant). Figure 2c,d show non-dimensional versions of Figure 2a,b, respectively.

From Figure 2 several immediate observations can be made. First, the exit velocity waveforms consist of long-time motions corresponding to glottal opening and closing. This behavior consists broadly of a rapid rise to a plateau early in the cycle, then an increase in speed as the glottis begins to close halfway through the time the glottis is open, and the flow has enough momentum to accelerate as the gap closes. This acceleration continues until roughly 0.75*T*_o_–0.8*T*_o_, when the jet speed rapidly drops to zero. Second, superimposed on these long-time motions are higher-frequency fluctuations which have been shown [1,2] to correspond to the passage of jet instability vortices through the exit plane.

Looking more closely at Figure 2, it can be seen that the rise to the plateau takes a larger fraction of the open time *T*_o_ as f* increases. Similarly, it can also be noted that the occurrence of the first sharp peak associated with vortex arrival at the glottis exit occurs later in the cycle, as f* increases. Since the first vortex arrives later in the cycle as f* increases, we note that, for the highest frequency cases, the arrival of the first vortex coincides with the jet velocity reaching the plateau level. In addition, in the middle of the cycle, the high-frequency fluctuations associated with jet vortex passage decrease, so that there is an interval of calm during which vortices do not form, until the flow accelerates later in the cycle.

**Figure 3.**
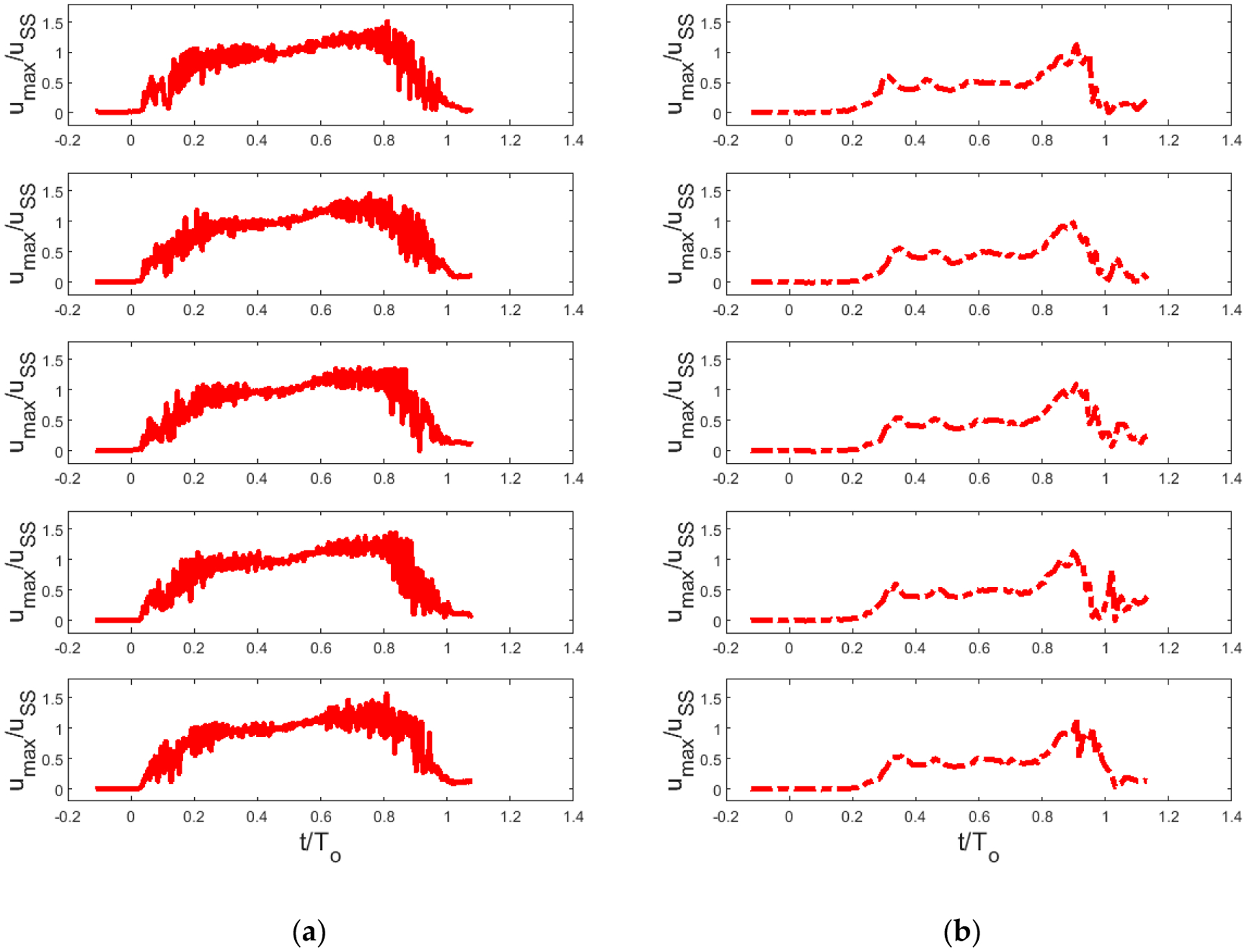
Glottal jet velocity waveforms, showing five realizations each, for lowest and highest reduced frequencies studied. (**a**) f* = 0.01, *Re*_h_ = 6560. (**b**) f* = 0.06, *Re*_h_ = 4120. Vortex motions are only partially phase-locked to vocal fold wall motions, giving rise to cycle-to-cycle variations in jet velocity, especially during glottal closure.

Focusing on Figure 2a,b, it can be observed that when *u*_SS_ is constant (Figure 2a), the time between vortex arrivals appears similar, while when *u*_SS_ is varied (Figure 2b), the time between vortex arrivals increases inversely proportion to *u*_SS_. Finally, over the range of *u*_SS_ and *T*_o_ studied, the fraction of the open time *T*_o_ occupied by a single vortex increases with f*. An important question regarding voice quality is whether these vortices sufficiently modulate glottal jet motions between cycles that each cycle produces a different sound. The data suggest this question can be answered affirmatively indication is shown in Figure 3, which shows five realizations each of exit jet speed waveforms for the lowest (f* = 0.01, *Re*_h_ = 6600, Figure 3a) and highest (f* = 0.06, *Re*_h_ = 4100, Figure 3b) reduced frequency cases. Each waveform is visibly different in terms of the arrival and amplitude of the glottal jet vortex peaks, especially during glottal closure. This question will be further addressed in the next section.

### 3.3. Calculating Jet Instability Vortex Formation Time

To quantify vortex timing, we compute the time of arrival of each sharp peak at the exit plane. To facilitate this computation, we first de-trend the exit velocity waveforms. This is accomplished by low-pass filtering each realization *u*_*n*_ to obtain:

(1) $$u_{\text{filt},n}=0.04\left(u_{\text{max},n-4}+u_{\text{max},n+4}\right)+0.08\left(u_{\text{max},n-3}+u_{\text{max},n+3}\right)+0.12\left(u_{\text{max},n-2}+u_{\text{max},n+2}\right)++0.16\left(u_{\text{max},n-1}+u_{\text{max},n+1}\right)+0.20u_{\text{max},n}.$$

Then, we compute the velocity fluctuation relative to the low-pass filtered velocity:

(2) $$u_{\text{fluc}}=u_{\text{max},n}-u_{\text{filt},n}$$

**Figure 4.**
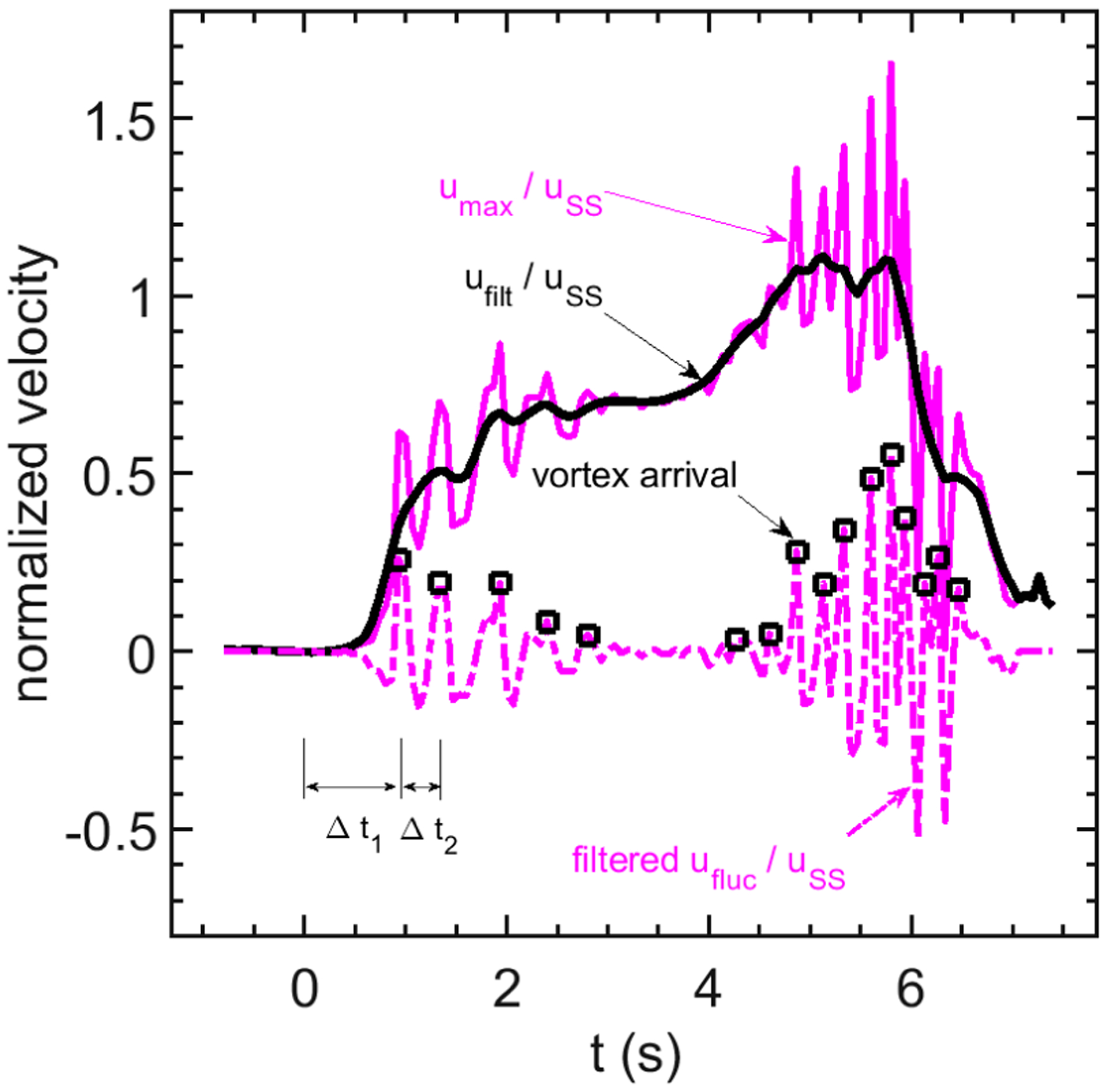
Example of signal treatment to obtain vortex formation times. –, Single realization of exit velocity waveform *u*_*n*_ for case *Re*_h_ = 7160, f* = 0.04; –, low-pass filtered waveform, ${\tilde{u}}_{n}$., –, velocity fluctuation *u*_*fluc*_ Peaks of *u*_*fluc*_ detected by *peakdetB.m* indicated by black squares. Vortex formation time estimates, Δ*t*_1_ and Δ*t*_2_, for the first two vortices are also indicated.

This analysis sequence is illustrated in Figure 4 for the case *Re*_h_ = 7200, f* = 0.04. The fluctuating velocity waveform *u*_fluc_ is then analyzed using a MATLAB script *peakdetB.m*, which computes the time index and amplitude of a signal at the local maxima and minima of a time series. For the example shown in Figure 4, the local maxima peaks found by this analysis are indicated by black squares. In this article, the maxima times *t*_*m*_ are used to compute the formation time for each subsequent vortex:

(3) $$\Delta t_{m}=t_{m}-t_{m-1}$$

Note that the formation time for the first vortex, Δ*t*_1_, is measured relative to the start of the cycle (*t* = 0 s).

**Figure 5.**
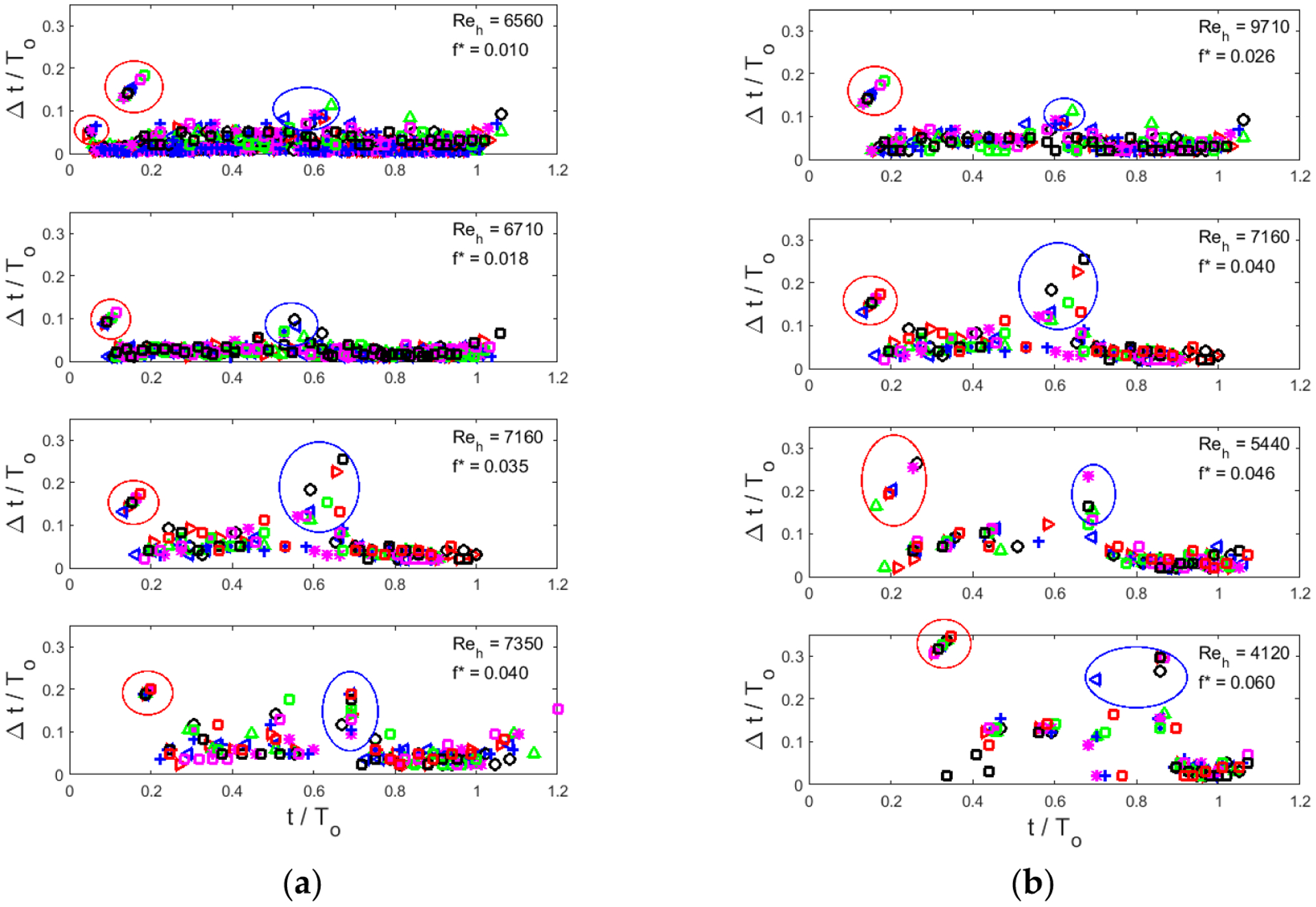
Vortex timing vs. cycle phase. (**a**) Constant *u*_SS_ cases, with *T*_o_ decreasing from top to bottom, resulting in nominally constant Reynolds number *Re*_h_, and increasing reduced frequency f*. (**b**) Constant *T*_o_ cases, with *u*_SS_ decreasing from top to bottom, resulting in decreasing Reynolds number *Re*_h_, and increasing reduced frequency f*. Note that the case *Re*_h_ = 7160, f* = 0.040 appears in both columns. Each realization is indicated by different symbol, to highlight differences in vortex formation times between cycles. Data points inside red circles correspond to the first vortex (leading the jet), and points in blue circles indicate the first vortex after the mid-cycle calm interval.

Figure 5 shows nondimensional vortex formation time, Δ*t*/*T*_o_, vs., cycle phase, *t*/*T*_o_, at which the vortex passes through the exit plane. Using this scaling presents vortex formation time in terms of its fraction of the vibration cycle period. Conditions for each case are denoted in the upper right corner of each plot. In general, four features of each plot are evident: (1) the initial vortex (circled in red in Figure 5), which leads the jet, generally takes the longest to form, (2) the time between subsequent vortices is longer early in the cycle than it is later in the cycle, and these two intervals are separated by the calm period discussed above, (3) the end of this calm period is indicated in Figure 5 by longer vortex separation times (circled in blue in Figure 5), and (4) there is a great deal of variation in vortex formation time between realizations. The last observation confirms that, even for this low number of realizations, phase coherence between glottal jet vortex behavior and glottal gap variation is weak (i.e., that any contributions of glottal jet vortices to sound production is broadband, as expected (see, e.g., [3,4,29]).

To consider Reynolds number and reduced frequency trends, examine first Figure 5a, in which the tunnel speed is held constant and the glottis open time is varied (i.e., the Reynolds number is essentially constant, but the reduced frequency increases by a factor of 4 from the top to the bottom plot). We note that the initial vortex pair that leads the glottal jet arrives later in the cycle as f* increases. (This trend does not apply entirely to the f* = 0.01 case. As can be seen in Figure 2a,d, this case shows a qualitatively different behavior in that the leading vortex often passes through the exit plane well before the jet velocity reaches its plateau level, unlike almost every other case studied.) A second trend is that the non-dimensional time between vortices increases, i.e., vortex formation time takes up a greater and greater fraction of the cycle period, as f* increases. It also appears that for f* > 0.01, the end of the calm period, during which no vortices pass through the glottis exit, occurs later and later as f* increases.

Assessing the trends for Figure 5b, where the open time *T*_o_ is held constant, but tunnel speed is varied, resulting in variation of both *Re*_h_ and f*, as indicated in each plot. The same trends with f* are observable as in Figure 5a: as f* increases, the initial vortex passes the exit later in the cycle, the calm period ends later in the cycle, and the time between vortices is longer before the calm period than after it.

## 4. Discussion

The results presented here bear on how the broadband portion of voice production occurs. First, it is clear that glottal jet instability vortices are not entirely phase-coherent with the vibration cycle, so they indeed provide a broadband component to voiced sound. Second, the glottal jet vortices display the greatest amplitude, and highest frequency, late in the vibration cycle, consistent with the observations of Hermes [5].

It should be noted that the high-amplitude, high-frequency vortex behavior, occurring during the late closure phase of the cycle, as observed here, can also contribute to vocal fold drag, though this will likely occur at lower frequencies than interactions with supraglottal anatomy, since the shape of the vocal fold is less “sharp” than, e.g., the epiglottis. While the current data cannot resolve the contributions to perceived broadband voiced sound from glottal jet forces on the vocal folds or supraglottal structures, both explanations are consistent with the data presented here and with Hermes’ observations [5]. In either case, the current data does suggest, however, that as f* increases, the high-frequency noise burst occurs later and later in the cycle.

A particularly interesting result from the data presented here is that the vortex formation time occupies a larger and larger fraction of the cycle period as reduced frequency f* increases. Even in the upper frequency range of the data presented here, equivalent to the upper range of adult male voices, vortex formation time is a considerable fraction of the cycle period. This suggests that, at a frequency higher than that in the data presented, vortex formation time becomes commensurate with the cycle period. This also suggests a possible aerodynamic regime change that may correspond to a change in voice register. It is also possible that such a regime switch contributes to the perceived difference between the adult male and female voices. These speculations motivate further research in this area.

## Figures and Tables

**Table 1.** Cases studied. Glottal jet velocity scale *u*_SS_ is the flow speed in the glottis with the glottis held open at maximum opening *h*_max_. Glottis open time *T*_o_ is the time glottis takes to open and close. f* is the reduced frequency of vocal fold vibration, *Re*_h_ the Reynolds number, *N* the number of realizations collected for each case, and *f*_life_ the equivalent life-scale frequency for each case.

*u*_SS_ (cm/s)	*T*_o_ (s)	*h*_max_ (cm)	f*	*Re*_h_	*N*	*f*_life_ (Hz)
28	23.7	2.34	0.010	6600	7	32
28	12.3	2.40	0.018	6700	10	61
28	6.53	2.56	0.035	7200	10	115
28	5.67	2.62	0.040	7300	10	132
16.1	6.53	2.56	0.060	4100	10	115
21.3	6.53	2.56	0.046	5400	10	115
38	6.53	2.56	0.026	9700	10	115
